# Improving eye care in Rwanda

**DOI:** 10.2471/BLT.14.143149

**Published:** 2015-04-30

**Authors:** Agnes Binagwaho, Kirstin Scott, Thomas Rosewall, Graeme Mackenzie, Gweneth Rehnborg, Sjoerd Hannema, Max Presente, Piet Noe, Wanjiku Mathenge, John Nkurikiye, Francois Habiyaremye, Theophile Dushime

**Affiliations:** a Ministry of Health of Rwanda, PO Box 84, Kigali, Rwanda.; b Interfaculty Initiative in Health Policy, Harvard University, Boston, United States of America (USA).; c Vision for a nation, Kigali, Rwanda.; d Adlens, Boston, USA.; e Fred Hollows Foundation, Rosebery, Australia.; f Christoffel Blinden Mission, Bensheim, Germany.; g Rwanda International Institute of Ophthalmology, Kigali, Rwanda.; h Dr Agarwal’s Eye Hospital, Kigali, Rwanda.

## Abstract

**Problem:**

Visual impairment affects nearly 285 million people worldwide. Although there has been much progress in combating the burden of visual impairment through initiatives such as VISION 2020, barriers to progress, especially in African countries, remain high.

**Approach:**

The Rwandan Ministry of Health has formed partnerships with several nongovernmental organizations and has worked to integrate their efforts to prevent and treat visual impairment, including presbyopia.

**Local setting:**

Rwanda, an eastern African country of approximately 11 million people.

**Relevant changes:**

The Rwandan Ministry of Health developed a single national plan that allows key partners in vision care to coordinate more effectively in measuring eye disease, developing eye care infrastructure, building capacity, controlling disease, and delivering and evaluating services.

**Lessons learnt:**

Collaboration between stakeholders under a single national plan has ensured that resources and efforts are complementary, optimizing the ability to provide eye care. Improved access to primary eye care and insurance coverage has increased demand for services at secondary and tertiary levels. A comprehensive strategy that includes prevention as well as a supply chain for glasses and lenses is needed.

## Résumé

**Problème:**

Près de 285 millions de personnes dans le monde présentent des déficiences visuelles. Bien que les efforts pour réduire la charge qu'elles représentent aient beaucoup progressé, grâce à des initiatives telles que VISION 2020, les obstacles au progrès, en particulier dans les pays d'Afrique, restent importants.

**Approche:**

Le Ministère de la Santé rwandais a établi des partenariats avec plusieurs organisations non gouvernementales en tâchant d'intégrer leurs efforts pour prévenir et traiter les déficiences visuelles, notamment la presbytie.

**Environnement local:**

Le Rwanda, un pays d'Afrique de l'Est comptant environ 11 millions d'habitants.

**Changements significatifs:**

Le Ministère de la Santé rwandais a mis en place un plan national qui permet une coordination plus efficace entre les partenaires clés de l'ophtalmologie, pour dépister les maladies des yeux, développer des infrastructures de prise en charge des affections oculaires, renforcer les capacités, contrôler les maladies, fournir des services et évaluer ces derniers.

**Leçons tirées:**

La collaboration entre les différents acteurs, dans le cadre d'un plan national unique, a permis d'assurer la complémentarité des ressources et des efforts et d'optimiser les capacités de prise en charge des affections oculaires. L'amélioration de l'accès aux soins oculaires de base ainsi qu'aux assurances a intensifié la demande de services aux niveaux secondaire et tertiaire. Une stratégie globale de prévention et d'acquisition de lunettes et de lentilles est maintenant nécessaire.

## Resumen

**Situación:**

La discapacidad visual afecta aproximadamente a 285 millones de personas en todo el mundo. Si bien se han hecho muchos avances en la lucha contra la carga de la discapacidad visual mediante iniciativas como el programa VISIÓN 2020, las barreras hacia el progreso siguen siendo altas, especialmente en los países de África.

**Enfoque:**

El Ministerio de Salud de Rwanda ha establecido colaboraciones con varias organizaciones no gubernamentales y ha trabajado para integrar sus esfuerzos para prevenir y tratar la discapacidad visual, incluida la presbicia.

**Marco regional:**

Rwanda, un país del África oriental con aproximadamente 11 millones de habitantes.

**Cambios importantes:**

El Ministerio de Salud de Rwanda desarrolló un único plan nacional que permite a los colaboradores clave que trabajan en la atención oftálmica coordinar de una manera más efectiva la medición de las enfermedades oculares, el desarrollo de una infraestructura para la atención oftálmica, la generación de capacidades, el control de las enfermedades y la prestación y evaluación de servicios.

**Lecciones aprendidas:**

La colaboración entre las partes interesadas bajo un único plan nacional ha garantizado que los recursos y esfuerzos sean complementarios, optimizando la capacidad de proporcionar servicios de atención oftálmica. La mejora del acceso a la atención oftálmica primaria y la cobertura del seguro ha incrementado la demanda de servicios a nivel secundario y terciario. Se necesita una estrategia exhaustiva que incluya la prevención y una cadena de suministro de gafas y lentes.

## ملخص

**المشكلة:**

يؤثر ضعف البصر على ما يقرب من 285 مليون شخص على مستوى العالم. وبالرغم من وجود تقدم كبير في التغلب على عبء ضعف البصر من خلال مبادرات مثل VISION 2020، فإن العوائق الموجودة أمام التقدم، خاصةً في البلدان الأفريقية، لا تزال مرتفعة.

**الأسلوب:**

لقد شكلت وزارة الصحة الرواندية مبادرات شراكة مع العديد من المنظمات غير الحكومية وعملت على إدماج جهودها للوقاية من ضعف البصر وعلاجه، بما في ذلك قصور البصر الشيخوخي.

**المواقع المحلية:**

رواندا، وهي بلد تقع في شرق أفريقيا ويسكن بها قرابة 11 مليون نسمة.

**التغيّرات ذات الصلة:**

قامت وزارة الصحة الرواندية بإعداد خطة وطنية منفردة تسمح للشركاء الرئيسين في مجال رعاية الإبصار بالتنسيق بشكل أكثر فعالية في قياس الأمراض العينية، وإعداد البنية التحتية للرعاية العينية، وإمكانية بناء القدرات، ومكافحة الأمراض، وتقديم الخدمات وتقييمها.

**الدروس المستفادة:**

لقد ضمن التعاون بين الجهات المعنية في إطار خطة وطنية منفردة أن تكون الموارد والجهود متكاملة، مما يؤدي إلى تحسين إمكانية توفير الرعاية العينية. أدى تحسين فرص الحصول على الرعاية العينية الأولية وتغطية التأمين على زيادة الطلب على الخدمات في المستويات الثانوية وفوق الثانوية. وهناك حاجة إلى استراتيجية شاملة تتضمن الوقاية بالإضافة إلى سلسلة الإمداد للنظارات والعدسات.

## 摘要

**问题:**

视力受损影响了全球近 28.5 亿人口。尽管我们已通过“视觉 2020”(VISION 2020) 等举措在消除视力受损带来的负担方面取得了很大的进步，但是前进的道路仍然充满障碍，这种情况在非洲国家尤为明显。

**方法:**

卢旺达卫生部已与多个非政府组织建立了合作伙伴关系，并且共同致力于预防和治疗视力受损，包括老花眼。

**当地状况:**

卢旺达是东非国家，拥有约 1.1 亿的人口。

**相关变化:**

卢旺达卫生部制定出全国统一的计划，允许与重要的视力保健合作伙伴一起在测定眼疾、开发眼科护理基础设施、培养能力、控制疾病以及提供和评估服务方面开展更加有效的合作。

**经验教训:**

利益相关者依据全国统一的计划而开展的合作能够确保资源和精力互补，以优化提供眼科护理服务的能力。初级眼科护理普及率提高以及保险覆盖面的改善增加了对中级和高级服务的需求。我们需要制定出一项涵盖预防举措和眼镜及镜片供应链的全面战略。

## Резюме

**Проблема:**

От нарушений зрения страдают около 285 миллионов человек во всем мире. И хотя благодаря таким инициативам, как VISION 2020, в борьбе с расстройствами зрения был достигнут немалый прогресс, на этом пути сохраняются значительные препятствия, особенно в странах Африки.

**Подход:**

Министерство здравоохранения Руанды в партнерстве с несколькими неправительственными организациями стремится объединить усилия этих организаций по профилактике и лечению нарушений зрения, включая старческую дальнозоркость.

**Местные условия:**

Руанда — восточноафриканское государство с населением около 11 миллионов человек.

**Осуществленные перемены:**

Министерство здравоохранения Руанды разработало единый национальный план, который позволяет ключевым партнерам в области офтальмологической помощи более эффективно координировать друг с другом работу по мониторингу нарушений зрения, разработке необходимой медицинской инфраструктуры, расширению своих возможностей, контролю заболеваний, а также оказанию услуг и оценке их качества.

**Выводы:**

Сотрудничество между заинтересованными сторонами в рамках единого национального плана позволило объединить их усилия и ресурсы для оказания оптимальной офтальмологической помощи. Благодаря упрощению процесса получения первичной офтальмологической помощи и страховому покрытию увеличился спрос на услуги на вторичном и третичном уровнях. Необходима комплексная стратегия, которая включала бы профилактику, а также учитывала бы цепь поставок линз и очков.

## Introduction

Visual impairment – both preventable and treatable – affects an estimated 285 million people globally. Most of the people affected (87%) live in low- and middle-income settings.^1^^,^^2^ In Africa, an estimated 32 700 people per million are visually impaired.^3^ A variety of eye disorders contribute to visual impairment, including cataract, glaucoma, trachoma and refractive error.^3^ Nearly 80% of impairments are preventable or treatable.^4^ Presbyopia – difficulty focusing on nearby objects – is a common feature of ageing, as the ocular lens loses elasticity. The ageing population and the lack of national plans to address the effect of visual impairment on people’s productivity and quality of life, will likely increase the burden of eye disease in many African countries.^5^

Providing eye care services to mitigate visual impairment is an important dimension of delivering comprehensive primary health care. It also contributes to economic growth and development by helping reduce injuries and by improving access to education and employment.^6^ Investment in eye care services has a benefit-to-cost ratio of more than two to one.^4^^,^^7^

While there is multilateral organizational support for integrating eye care services into health systems – including the launch of the VISION 2020 Right to Sight Initiative – there are barriers to progress, especially in low-income settings.^2^ In many African countries, there is a shortage of eye care personnel, a lack of standardized training, inadequate coordination among eye care stakeholders and for those in need of eye care, the cost of equipment and treatment can be an obstacle.^4^^,^^8^ Challenges with data collection and measurement of the burden of visual impairment complicate efforts to generate support for effective health policy development.^8^ Here we summarize ongoing efforts to overcome barriers to addressing the burden of visual impairment in Rwanda.

## Setting

Rwanda has 10.5 million inhabitants. Over the past two decades, mortality caused by infectious diseases has dropped, for instance, mortality related to acquired immunodeficiency syndrome fell by 82% between 2000 and 2012. Consequently, life expectancy has nearly doubled since the 1990s and is now at 63 years of age.^9^ In the population older than 49 years, in 2006, the overall prevalence of visual impairment was 5.3% (not including presbyopia) and the prevalence of blindness was 1.8%.^10^ In 2006, more than 80% of the eye conditions were considered preventable or treatable – including cataract, refractive error and trachoma.^10^ Other estimates suggest that more than 65 000 people (0.6%) in Rwanda are blind in both eyes and 12% of the population – including those with presbyopia – have a correctable refractive error and are therefore in need of corrective lenses.^11^

While Rwanda has experienced substantial economic growth, nearly 50% of the population still lives below the poverty line, especially in rural areas. This means that most people are not able to afford private eye care services.^12^ Moreover, most eye care resources are located in the capital of Kigali, a situation which has resulted in public–private partnerships aiming to ensure equity in access to eye care services.

## Forming partnerships

In 2002, Rwanda signed the VISION 2020 initiative and created a national vision plan to end needless blindness. The plan has been updated regularly in collaboration with stakeholders.^11^ As part of the plan, the Ministry of Health partners with various nongovernmental organizations (NGOs) and private providers to address the preventable and treatable burden of visual impairment, especially at the primary care level. Here we describe partnerships between the Ministry of Health and three international non-profit partners: Vision for a Nation, the Christoffel Blinden Mission and the Fred Hollows Foundation. We categorize these partnerships in four pillars: (i) measurement of disease prevalence and evaluation of services (e.g. funding disease burden studies); (ii) infrastructure development (e.g. building of eye care clinics); (iii) human resources development (e.g. standardizing the eye care curriculum for nurses); and (iv) disease control and service delivery (e.g. providing low-cost or free eye glasses to those in need; Table 1).

**Table 1:** Eye care in Rwanda: key functions of nongovernmental collaborators

Partnering organization	Start of partnership		Description of partnership	Support pillar			
				Measurement of disease burden and evaluation of services	Infrastructure development	Human resources development	Disease control and service delivery
Vision for a Nation		2010	An NGO dedicated to nationwide primary eye care for rapid provision of vision assessments and affordable eye glasses and referral capabilities. The NGO works in countries where there are limited or no such services and products available to most of the population.	Conducts on-going research on the provision of eye glasses and productivity of beneficiaries.^13^ Supports the monitoring of referrals and provision of eye glasses.		Incorporated training curriculum in all eight national nursing schools; educates CHWs to enable public access of treatment at local health centres; has trained primary eye care nurses in each of the 502 health centres to provide vision assessments, to dispense medications and eye glasses and to refer more complicated cases to hospital.	Introduced affordable eye glasses at all 502 health centres; mobilizes district level stakeholders and runs national radio campaigns to promote services; supports evaluations of the uptake of new primary eye care services; created a model for CHW training designed to raise awareness about new primary eye care services.
Fred Hollows Foundation		2006	An NGO that seeks to eradicate avoidable blindness in vulnerable populations and in resource-poor settings around the world.	Funded the first MoH rapid assessment of avoidable blindness survey in 2006 in the western province.^10^ Plans to fund the 2015 survey to update and compare to 2006 baseline results. Evaluated primary eye care training in the western province.	Built or refurbished three district eye units. Supports 12 district eye units for establishment of referral and capacity building that links with the primary level PEC services. Donated ophthalmic equipment to eight district eye units.	Proposed scholarship for postgraduate studies in ophthalmology for two practitioners per year. Supported the training of 1855 CHWs in eye care and blindness prevention between 2007 and 2013. Contributes funding to the College of Health Sciences to train 20 new mid-level eye care workers each year. Funded the development of the primary eye care curriculum.	60 000 people received eye care consultations in eye unit of health centres supported by FHF. Supported 2930 cataract operations. Supports the maintenance of ophthalmic equipment across the country.
Christoffel Blinden Mission		1993	An international disability and development organization committed to improve the quality of life for persons with disability and those at risk of disability in low-and middle-income countries.	Financed a study on vernal keratoconjunctivitis in 2007.	Sponsors the Catholic referral centre for eye health located in Kabgayi District Hospital in Muhanga district. Constructed the eye unit in Kabgayi and provided equipment.	Started the ophthalmic clinical officers course at Kigali Health Institute. Provides scholarships to train Rwandan ophthalmologists abroad and ophthalmic clinical officers at Kigali Health Institute. Kabgayi Eye Unit is a practical training centre for ophthalmic clinical officers and ophthalmologists in training.	Sponsors staff and materials needed to perform most eye operations in Rwanda. Since 2009, the Kabgayi team has provided approximately 4000 eye operations and 50 000 consultations yearly. The Kabgayi Eye Unit sponsors paediatric ophthalmology, vitreoretinal surgery and retinoblastoma treatment.

CHW: community health worker; FHF: Fred Hollows Foundation: MoH: Ministry of Health; NGO: nongovernmental organization; PEC: primary eye care.

Note*:* This table summarizes key international non-profit partners involved in eye care in Rwanda and is not exhaustive of all stakeholders involved in providing eye care services in Rwanda.

## Guiding principles

Three principles have guided these changes in the way eye care services are delivered in Rwanda: prioritizing geographic equity of service delivery, reducing the cost of access to services, and coordinating all partners under a single national plan.

**Fig. 1:**
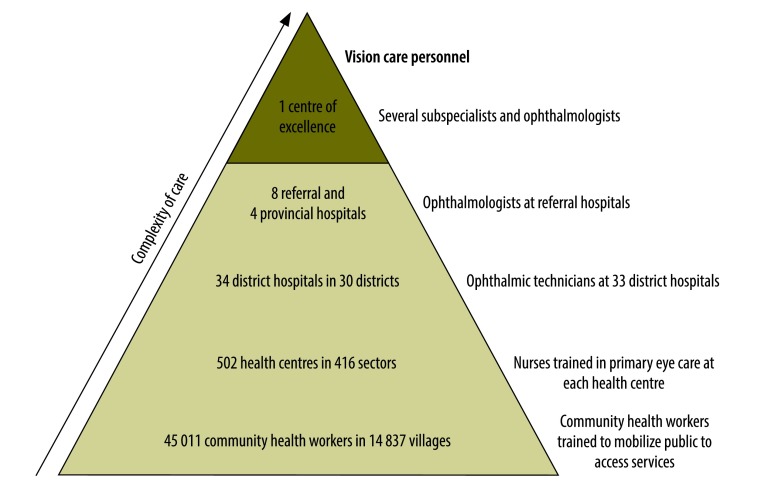
Structure of the health sector and organization of current vision care personnel in Rwanda

The Rwanda health system aims to provide eye care services at primary, secondary and tertiary levels **(**Fig. 1**)** in a decentralized and evenly distributed manner. Primary health care is delivered through a network of 45 000 community health workers and 502 health centres. In 2010 the Rwandan government launched a comprehensive primary eye care programme with the support of the organization Vision for a Nation. This programme includes creating a permanent primary eye care curriculum at all eight nursing schools in Rwanda, educating 1250 existing health centre nurses in primary eye care services and improving referral guidance. To date, the programme has administered approximately 200 000 vision assessments nationally and created a sustainable nationwide supply chain for eye glasses. National campaigns are run to inform people that they can access these services.

To ensure that services are affordable, the Rwandan community-based health insurance scheme, which enrols most of the population,^9^ now includes vision care services, including reimbursement for consumables.

The Ministry of Health coordinates partners by ensuring that their activities align with the national vision plan. A technical working group – consisting of partners and Ministry of Health representatives – advises on implementation. The working group provides planning and ensures that each partner is providing the most appropriate services, given their available resources and expertise. For example, through the primary eye care programme, efforts to increase awareness and attention to vision care at the primary level have increased demand for more advanced vision care at the secondary and tertiary care levels, including cataract surgery. Therefore, other partners – such as the Fred Hollows Foundation and the Christoffel Blinden Mission – have provided their expertise to create curricula, support scholarship programmes to train eye-care specialists, deliver specialty care and develop critical health-care infrastructure at these more resource-intensive levels. These efforts complement private eye care clinics and hospitals that also provide more advanced eye care services in Rwanda.

## Next steps

Although these non-profit collaborations have helped to orchestrate the development of eye care delivery infrastructure, human resource capacity and quantity of services, formal assessments will be necessary to document improvements in population health. Evaluation of the primary eye care programme is ongoing and the next population survey to assess the burden of avoidable blindness will be done this year. However, evidence from the Rwandan electronic health management information system suggests that demand for eye care services may be increasing: eye disease was the second leading reason for seeking care in 2014. In 2009, eye diseases were not among the 10 leading reasons for seeking care. Additional indicators – such as the number of cataract operations done and the number of people presenting with glaucoma – recently added to the electronic information system will help to monitor progress and identify remaining gaps, especially for more advanced eye care.

The World Health Organization has set a target of 2000 operations per million population per year for cataract surgery in Africa. The cataract surgery rate in Rwanda was estimated at 300 operations per million population per year in 2007.^14^ Also, despite the gradual increase in human capacity, there continues to be a shortage of trained eye care specialists across sub-Saharan Africa.^8^ In 2014, there were only 18 ophthalmologists in Rwanda, most of whom resided in the capital, leaving rural areas underserved.^10^ Policies to promote task shifting, such as through the Rwandan three-year ophthalmic technician training course, has helped to address this gap, yet more trained professionals will be needed. Other east African countries, such as Uganda, are also investigating task shifting to bolster workforce capacity through its ophthalmic clinical officers’ programme.^15^

Collaborations between the Ministry of Health and key partners have improved the capacity to manage visual impairment in Rwanda (Box 1). Though advanced eye care services need more development, we are optimistic that continued collaboration will provide opportunities to overcome the remaining challenges.

Box 1 **Summary of main lessons learnt**

- Collaboration between stakeholders under a single national plan has ensured that resources and efforts are complementary, optimizing the ability to provide eye care.
- Improved access to primary eye care and provision of insurance has increased demand for eye care services at secondary and tertiary levels.
- A comprehensive strategy is needed; one that includes prevention of eye disease and a supply chain for glasses and lenses.
